# Foreign Body in the Air Passages—Spontaneous Extrusion after 108 Days

**Published:** 1863-03

**Authors:** John Swain

**Affiliations:** Ballardsville, Ky.


### ORIGINAL COMMUNICATIONS.

### FOREIGN BODY IN THE AIR PASSAGES—SPONTANEOUS EXTRUSION AFTER 108 DAYS.

By JOHN SWAIN, M. D., Ballardsville, Ky.

On the morning of Oct. 5, 1862, I was called in haste to see Oscar Barnhill, aged about 9 years. The messenger saying he had swallowed a tack. On arriving at the house, the little patient informed me, that having the tack in his mouth, while looking up at a map upon the wall, he swallowed it; that he felt it scratch in its passage, all the way to the pit of his stomach.

He had already vomited on the hearth, but was confident he had not thrown it up, for he still felt it in his stomach. I caused him to swallow a raw egg, and in some ten minutes gave him an emetic. In the mean time, I manipulated and auscultated the trachea and examined the chest, believing that it might be lodged there rather than the stomach; I discovered a wheezing in the upper portion of the trachea, extending a little below the cricoid; was informed that he had a cough for some days; he coughed occasionally, but not as frequently as he should with a foreign substance in his "windpipe;" pressure upon the trachea gave no evidence of its location. He vomited some four or five times, gave up his breakfast, and, I believe the entire contents of the stomach, but no tack. I again auscultated for some sign, but found nothing more than the wheezing as above. I sent for my friend, Dr. Wells, determining to perform laryngotomy, if upon canvassing the symptoms, we could fix the point of lodgment within surgical reach.

By the time of the Doctor's arrival, he had recovered from his fright and the effects of the emetic, and there being little or no disturbance in his respiration more than the cold might produce, after another careful examination, we concluded the child might be correct in his relation of its passage to his stomach, and that he left it in the ashes with the first emeses. We consequently (and wisely, as the result proves,) determined not to interfere, but leave the doubtful matter to nature, as the safest course. He immediately got up and went about his usual amusements.

He attended school from that time regularly, with no inconvenience, save a slight hacking cough night and morning, and an occasional wheezing upon extra exertion, (from which he was relieved by a few moments' rest,) up to the morning of the 26th day of Jan., 1863, 108 days from the time of inception, when, while in bed, by a violent fit of coughing, he expelled upon the bed-clothing, the identical tack, enveloped in bloody sputa; calling to his father, "here it is, here's the tack."

I have it in my possession, a hoe shaped saddler's tack, 6½ lines in length, with a head measuring upon its face 2½ by 2 lines; I say hoe shaped, the shaft being attached to one side of the head.

If the above hastily written narrative should appear to you worthy, you are at liberty to use it, and attach such remarks as may suggest themselves.

